# Effect of allosteric inhibition of non-muscle myosin 2 on its intracellular diffusion

**DOI:** 10.1038/s41598-020-69853-8

**Published:** 2020-08-07

**Authors:** Ádám I. Horváth, Máté Gyimesi, Boglárka H. Várkuti, Miklós Képiró, Gábor Szegvári, István Lőrincz, György Hegyi, Mihály Kovács, András Málnási-Csizmadia

**Affiliations:** MTA-ELTE Motor Pharmacology Research Group, Department of Biochemistry, Eötvös Loránd University, Pázmány Péter sétány 1/c, 1117 Budapest, Hungary

**Keywords:** Fluorescence imaging, Time-lapse imaging, Motor protein function, Myosin, Stress fibres

## Abstract

Subcellular dynamics of non-muscle myosin 2 (NM2) is crucial for a broad-array of cellular functions. To unveil mechanisms of NM2 pharmacological control, we determined how the dynamics of NM2 diffusion is affected by NM2′s allosteric inhibitors, i.e. blebbistatin derivatives, as compared to Y-27632 inhibiting ROCK, NM2′s upstream regulator. We found that NM2 diffusion is markedly faster in central fibers than in peripheral stress fibers. Y-27632 accelerated NM2 diffusion in both peripheral and central fibers, whereas in peripheral fibers blebbistatin derivatives slightly accelerated NM2 diffusion at low, but markedly slowed it at high inhibitor concentrations. In contrast, rapid NM2 diffusion in central fibers was unaffected by direct NM2 inhibition. Using our optopharmacological tool, Molecular Tattoo, sub-effective concentrations of a photo-crosslinkable blebbistatin derivative were increased to effective levels in a small, irradiated area of peripheral fibers. These findings suggest that direct allosteric inhibition affects the diffusion profile of NM2 in a markedly different manner compared to the disruption of the upstream control of NM2. The pharmacological action of myosin inhibitors is channeled through autonomous molecular processes and might be affected by the load acting on the NM2 proteins.

## Introduction

Stress fibers are contractile actomyosin bundles in non-muscle cells, playing fundamental roles in diverse biological functions^1–4^. Stress fibers and stress fiber-like structures play central roles in cell division as key components of the cleavage furrow^5^, determine cancer cell motility during metastasis^6^, influence cancer cell growth^7^, and they have been demonstrated as crucial elements in neuronal plasticity including neurite outgrowth^4,8–11^. These force-generating structures consist of actin filaments and different co-assemblies of non-muscle myosin 2 (NM2) isoforms^12,13^. The two major stress fiber-forming myosin isoforms in human cells are NM2A and NM2B, that together build up mixed minifilaments of various length and thickness^14^. Stress fibers may be categorized as central and peripheral, based on their subcellular localization. Central stress fibers include those localized ventrally, dorsally, in transverse arcs and those associated around the nucleus. Their main roles are in cell adhesion, motility, cell division and cell shape determination^15,16^. Peripheral stress fibers are located at the edge of the cell. Laser nanosurgery experiments have shown that incision of peripheral stress fibers causes them to retract significantly, suggesting that these structures are under larger mechanical strain than their central counterparts^17^. Resisting loads on NM2 heads have been shown to significantly decrease the rate of ADP release from actin-bound myosin heads, resulting in slower actomyosin dissociation^18–20^. NM2 minifilaments are also activated or repressed by the phosphorylation or dephosphorylation of the myosin light chains (MLC), respectively^21–24^. Rho-dependent kinase (ROCK) positively regulates NM2 by directly phosphorylating MLC and inhibiting MLC phosphatase in the central region of the cells^25,26^. If the kinase activity of ROCK is inhibited, MLC kinase (MLCK) and Zipper-interacting protein kinases substitute for direct ROCK activity in peripheral stress fibers, while ROCK retains its regulating effect on MLC phosphatase^24,25^. Also, regulation via the Rho pathway is more prominent in the case of the central fibers, while peripheral stress fibers fall under regulation by the CaM-Ca-MLCK system^21,22^. However, the two pathways are strongly connected via MLCP-MBS (myosin binding subunit) phosphorylation^27^. Furthermore, both stress fiber populations are affected by ROCK inhibition but with different sensitivity. Dephosphorylation of the regulatory MLC deactivates NM2 via triggering the formation of the inactive 10S state by shifting the equilibrium in favor of this state^25,28,29^. In the 10S state of NM2 the two heads interact with each other and with the tail domain of the same myosin molecule^28,29^. In this compact form NM2 loses its actin binding and ATPase activity and diffuses in the cell with high mobility^21,22^. This highly mobile form of NM2 allows for its rapid intracellular redistribution, which is otherwise unattainable by active translocation of NM2 assemblies^30–32^. A potent way to develop the 10S NM2 state in cells is by treating the cells with the ROCK inhibitor Y-27632^33,34^. This inhibitor promptly blocks ROCK activities and quickly results in a loss of stress fiber integrity^22^. In line with the spatial distribution of ROCK activity, central stress fibers were disrupted shortly after Y-27632 treatment, while peripheral stress fibers were less affected in cultured fibroblasts^25^.

In contrast to dephosphorylation that facilitates the formation of the 10S state, NM2 ATPase activity can be directly inhibited by allosteric myosin inhibitors without the formation of 10S state. Blebbistatin^35^, a potent allosteric inhibitor of NM2, has been shown to stabilize the posthydrolytic (ADP.P_i_) state of myosin, thereby blocking the ATPase cycle. In this state myosin heads are weakly bound to actin^36^. Blebbistatin inhibits the ATPase and actin filament sliding activities of all stress fiber-forming NM2 isoforms^35,37–39^. However, adverse properties of blebbistatin have hindered its application in cell biological and in vivo experiments. The low solubility of blebbistatin precludes its application at effective and precise concentrations in cells due to the formation of aggregated precipitates in cell cultures or animal tissues. Elevated concentrations of blebbistatin are highly cytotoxic to mammalian cells; thus, time dependent processes could not be reliably measured. Moreover, blebbistatin is highly fluorescent when 480 nm excitation is applied, which seriously limits its use when a GFP-tagged protein is investigated. Similarly, blebbistatin’s intrinsic fluorescence also interferes with fluorescence recovery after photobleaching (FRAP) experiments^40^. These features together make the analysis of visualization-based experiments in the presence of blebbistatin cumbersome and at least ambiguous. However, we recently developed a new blebbistatin derivative, para-Nitroblebbistatin (pNbleb), which obviates most of the disadvantageous properties of the original molecule^41^. We have also introduced para-Aminoblebbistatin (AmBleb), which has greatly increased solubility and minimal fluorescence^42^. Both of these new derivatives retain the inhibitory properties of blebbistatin, but can be effectively and safely applied in in vivo experiments. We further refined our experiments using our recently developed optopharmacological technique, Molecular Tattoo, which uses two-photon microscopy to covalently crosslink a photoreactive blebbistatin derivative, azidoblebbistatin (N_3_Bleb) to the enzyme in any selected subcellular area of interest, down to a sub-femtoliter volume^43^. As activation by scanning with the two-photon microscope can be repeated multiple times in the same area, the effective concentration of the bound molecules can be gradually raised. This allows the photo-crosslinkable compound to be applied in a sub-effective concentration, while still reaching effective concentration of the covalent enzyme-inhibitor complex in the tattooed area. Thus, Molecular Tattoo is capable of confining the effects of a drug to subcellular compartments while avoiding systemic effects.

Combining the above developments, in the present study we were able to examine the effects of allosteric inhibition of NM2 on its diffusion dynamics both in a whole cell and localized to a stress fiber. We compared these results with the effects of dephosporylation-induced inactivation of NM2. Do NM2 trapping during its ATPase cycle by blebbistatin derivatives, versus inactivating it by dephosphorylation via ROCK inhibition, have a different effect on its diffusion in the cell? We find that treatment with Y-27632 and blebbistatin derivatives result in markedly different stress fiber dynamics. We localized the effects of blebbistatin to the central and peripheral stress fibers separately, and were able to reproduce the effects of blebbistatin derivatives in a targeted peripheral region without affecting the rest of the cell. Our results provide a strong indication that Y-27632 and blebbistatin treatment should not be considered as mechanistically identical in cell physiological experiments. Furthermore, with the help of the Molecular Tattoo technique, the effects of N_3_Bleb remain localized to the bleached area on a single stress fiber, which indicates an autonomous mechanism by which blebbistatin exerts its effects on fiber dynamics.

## Results

### NM2 inhibition results in stress fiber destabilization

**Figure 1:**
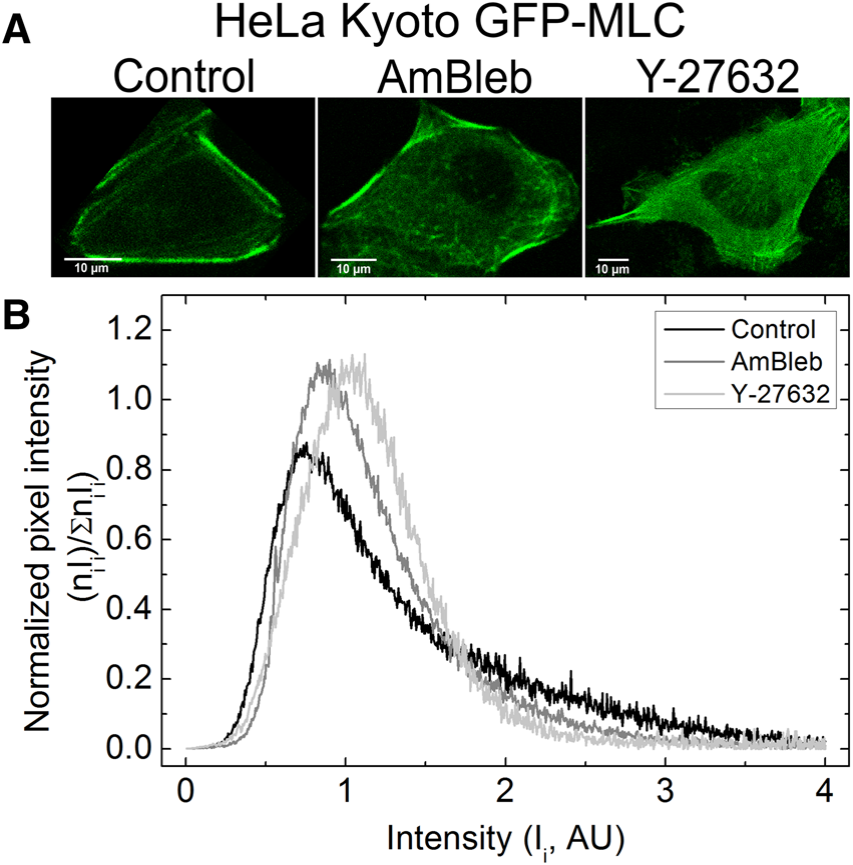
Destabilization of peripheral stress fibers by AmBleb and Y-27632. (**A**) GFP-labeled MLC (GFP-MLC) expressing HeLa Kyoto cells were imaged in two-photon microscope in the absence (n = 10) and presence of 40 μM AmBleb (n = 10) or 2 μM Y-27632 (n = 10). Control cells showed that high-intensity peripheral stress fibers remained intact after 30 min in the absence of inhibitors. (**B**) Normalized pixel intensity distribution histograms (n = pixel frequency; I = intensity), revealing changes resulting from inhibitor treatment (see text).

HeLa Kyoto cells were treated with AmBleb or Y-27632 in order to investigate the effect of different inhibitory mechanisms on the subcellular distribution of NM2-containing structures. GFP-MLC expressing cells (rat myosin light chain 12B labeled with GFP (MLC12B)^44^) were imaged using a scanning mode two-photon microscope after incubating the cells with either AmBleb (40 µM) or with Y-27632 (2 µM) (Fig. 1A). The concentration of Y-27632 was chosen based on the work of Ruiz-Loredo et al. which utilized a similar stress fiber disrupting property of the inhibitor^45^. The cited work used 0.5, 1 and 5 µM of the inhibitor; however, we found that employing 2 μM of the inhibitor was sufficient to dissolve stress fibers, while at this concentration we could minimize the aspecific effects of the inhibitor. The background signal of the images (fluorescence outside cells) was subtracted, and the normalized distributions of pixel intensities were obtained. The intensity profiles were normalized to aid comparison between intensity distributions recorded under different treatment conditions (Fig. 1B). Using two-sample t-test we compared the normalized pixel intensity distributions, which showed significant differences between the control and the inhibitor-treated distributions (*p*_Contr vs AmBleb_ = 6.3 × 10^−6^; *p*_Contr vs Y-27632_ = 2.3 × 10^−4^); however, the comparison of distributions for Y-27632 and Ambleb treated cells yielded no significant difference (*p*_Ambleb vs Y-27632_ = 0.13). These results indicate that AmBleb and Y-27632 treatments alter the stress fiber dynamics and cause the GFP-tagged NM2 molecules to redistribute across the imaging plane (and presumably across the whole volume of the cell). In earlier studies, a similar effect of Y-27632 was indicated in fibroblasts^25^. These results suggest that the applied drugs can cause NM2 minifilaments to detach from the stress fibers. The notable difference in the shape of the pixel intensity distribution of Y-27632, as compared to AmBleb, may reflect the different mechanisms of action of the two inhibitors on stress fiber dynamics. Accordingly, experiments using phalloidin staining showed disruption of actin bundles in cells treated with Y-27632, while for AmBleb the actin fibers remained mainly intact (Supplementary Fig. 1). Experiments performed with cells containing GFP-tagged NM2 heavy chain constructs yielded similar results (Supplementary Fig. 2), as well as those for cells stained with an actin-specific dye (Supplementary Fig. 3).

### NM2 diffuses faster in central fibers than in peripheral fibers in the absence of inhibitors

**Figure 2:**
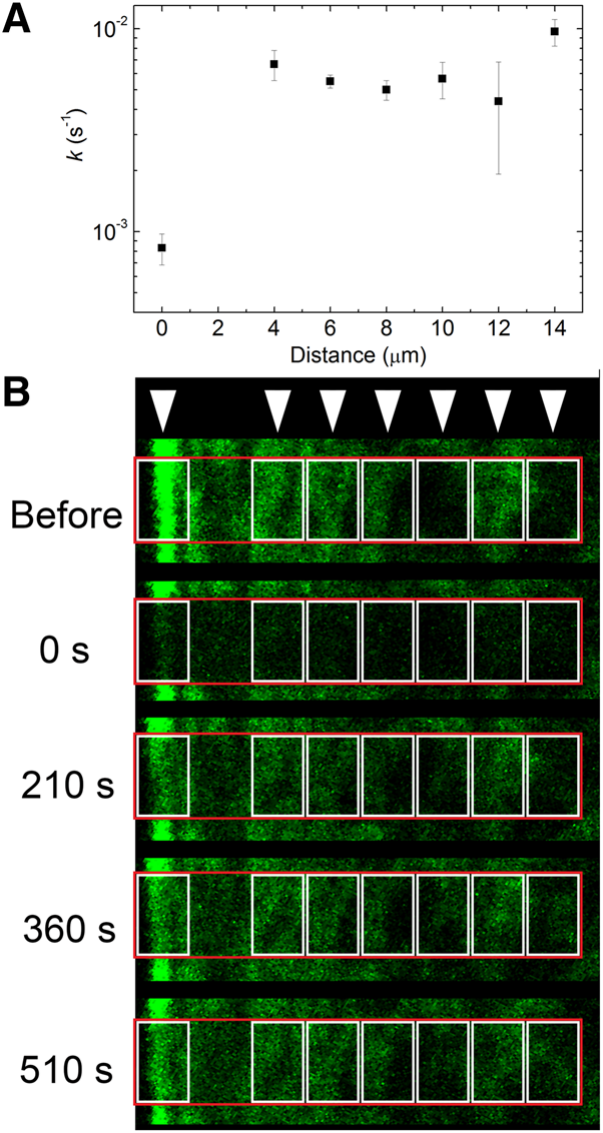
Peripheral stress fiber dynamics is an order of magnitude slower than that of central fibers in the absence of inhibitors (see also Supplementary Video 1 and 2). (**A**) Rate constants of fluorescence recovery, plotted as a function of distance from the peripheral stress fiber (means ± SEM, *n* = 3). The recovery rates of the peripheral stress fibers were an order of magnitude slower than those of central fibers. (**B**) Sample images at different time points from a FRAP experiment in a GFP-MLC expressing HeLa Kyoto cell, where a 4 × 14 μm region (red box), including both peripheral and central stress fibers, was photobleached in the absence of NM2 inhibitors. Fluorescence recovery was analyzed in the regions indicated by white arrows and white boxes, corresponding to the abscissa of panel **A**.

NM2 diffusion in GFP-MLC expressing HeLa Kyoto cells was investigated by FRAP experiments. Peripheral and central stress fibers were simultaneously photobleached in a 4 × 14 μm region and rate constants of fluorescence recovery were determined in different regions of the photobleached area (Fig. 2, Supplementary Videos 1, 2). We found that the rate constants in the central fibers were one order of magnitude higher compared to those in the peripheral stress fibers, indicating a large difference in NM2 diffusion between the two types of stress fibers. Furthermore, these tests have shown that the FRAP recovery rate of the central stress fibers does not vary significantly with the distance from the cell periphery. Upon testing the dependence of the recovery rate on initial, un-bleached stress fiber intensity, we found that there is no correlation between these parameters (Supplementary Fig. 4).

### ROCK inhibition accelerates peripheral stress fiber diffusion while blebbistatin derivatives suppress stress fiber dynamics at saturating concentrations

**Figure 3:**
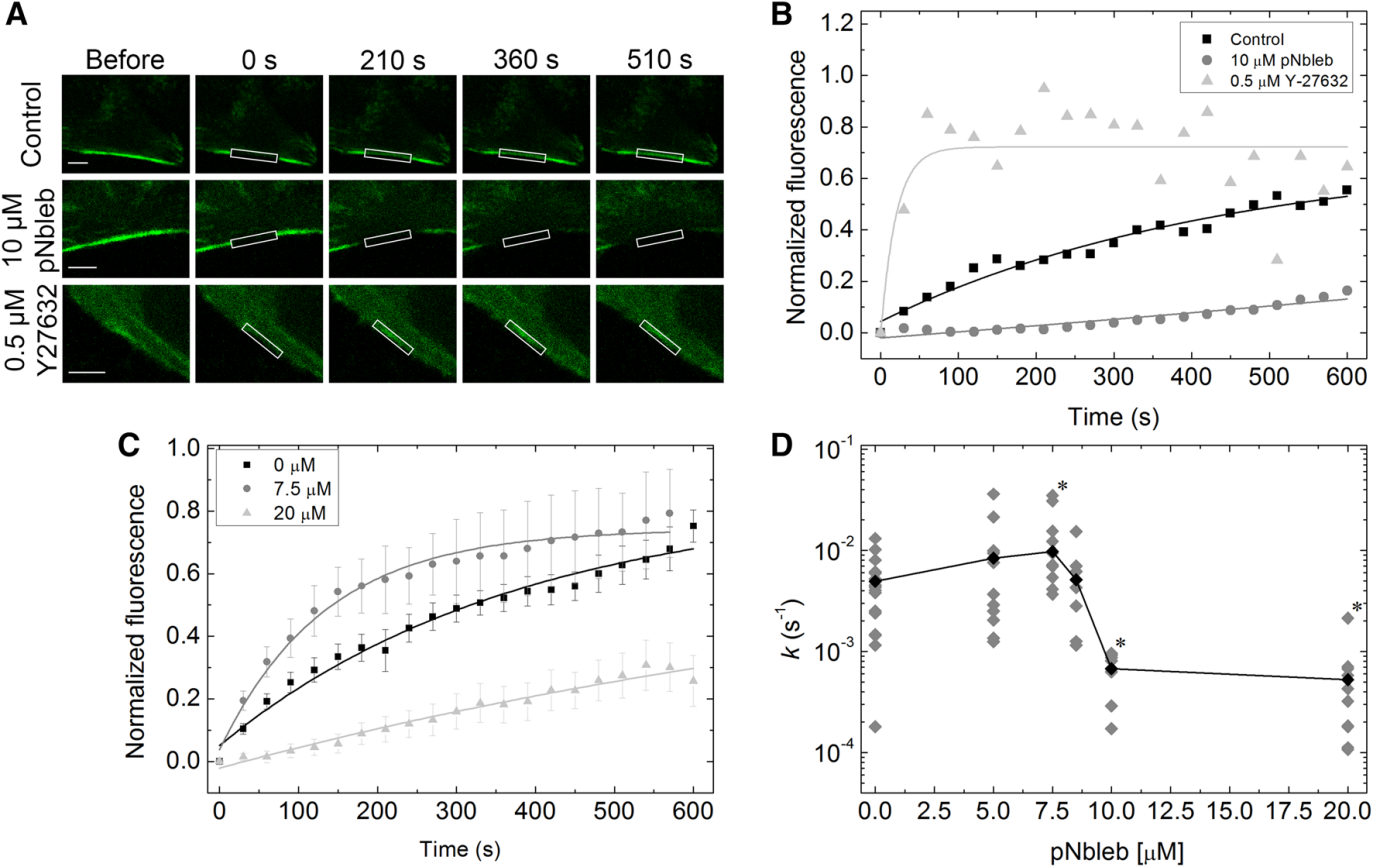
FRAP recovery rates show marked difference between Y-27632 and pNbleb inhibition in peripheral stress fibers (see also Supplementary Video 3). (**A**) Representative FRAP images of peripheral stress fibers in GFP-MLC expressing HeLa Kyoto cells at different time points in the absence and presence of 10 μM pNbleb or 0.5 μM Y-27632. White boxes indicate the photobleached areas. Scale bar: 5 μm. (**B**) Fluorescence levels normalized to the pre-photobleaching level are plotted as a function of time after photobleaching. Fluorescence recovery rate constants determined from single exponential fits showed significant differences in peripheral stress fiber dynamics in the presence of the different inhibitors (mean values: *k*_contr_ = 2.3 × 10^−3^ ± 7.4 × 10^−4^ s^−1^ (black), *k*_pNbleb_ = 2.9 × 10^−4^ ± 2.8 × 10^−3^ s^−1^(gray), *k*_Y27632_ = 5.3 × 10^−2^ s^−1^ ± 1.8 × 10^−4^ s^−1^ (light gray)). (**C**) Averaged fluorescence recovery of peripheral stress fibers recorded in the presence of 0 μM, 7.5 μM and 10 μM pNbleb (means ± SEM, sample sizes as specified for panel **D**). Single exponential fits are shown as light grey, grey and black lines, respectively. (**D**) Plot of the fluoresce recovery rate constants as a function of pNbleb concentration (n_0 μM_ = 18, n_5 μM_ = 11, n_7.5 μM_ = 12, n_8.5 μM_ = 7, n_10 μM_ = 8, n_20 μM_ = 10)). The graph shows all acquired data points. The datasets marked with black asterisks are significantly different from the control recovery rates (0 μM), according to parametric ANOVA tests (see “Materials and methods”). Fitted parameters of individual FRAP tests and their averages are shown as gray and black diamonds, respectively.

As shown above, peripheral stress fiber structure is only slightly affected by AmBleb or pNbleb treatments, whereas it is almost completely dissolved by Y-27632 (Figs. 1A and 3A). To distinguish the effects of Y-27632 and a blebbistatin derivative (pNbleb) on peripheral stress fiber dynamics, we performed FRAP experiments on HeLa Kyoto GFP-MLC cells in the presence of either inhibitor (Fig. 3A,B). Photobleaching of the high load-bearing peripheral stress fibers and the subsequent monitoring of fluorescence recovery were carried out using two-photon microscopy (Fig. 3A). The concentration of Y-27632 had to be decreased to 0.5 µM in these experiments in order to find an at least partially retained stress fiber to be photobleached. Measured fluorescence intensities were normalized to the maximum fluorescence level of GFP before bleaching. Normalized data points were fitted with a single exponential function (Fig. 3B). We found that the fluorescence recovery rate constant in the presence of Y-27632 was 20 times faster than that in untreated cells (*k*_Y27632_ = 5.3 × 10^−2^ s^−1^, *k*_contr_ = 2.3 × 10^−3^ s^−1^, indicating the presence of a highly mobile NM2 population. In contrast, in the presence of 10 µM pNbleb, fluorescence recovery was an order of magnitude slower than that in the untreated cells (*k*_pNbleb_ = 2.9 × 10^−4^ s^−1^) (Fig. 3B). The increased recovery rates in the Y-27632 experiments suggest a shift in equilibrium toward the inactive but highly mobile 10S state of NM2. In contrast, pNbleb treatment slows recovery, suggesting that pNbleb affects NM2 diffusion in a different way.

### Peripheral stress fiber dynamics is accelerated at low pNbleb concentrations and suppressed at high pNbleb concentrations

To further investigate the effects of the inhibition of the ATPase activity of NM2 on peripheral stress fiber dynamics, we measured the pNbleb dose dependency of FRAP rate constants (Fig. 3C,D, Supplementary Video 3). FRAP experiments were carried out using HeLa Kyoto GFP-MLC cells in the presence of different concentrations of pNbleb (0–20 µM, Fig. 3C). Normalized fluorescence data points were plotted as a function of time and fitted with single exponential functions (Fig. 3C and Supplementary Fig. 5). Strikingly, the rate constants did not depend on pNbleb concentration monotonically (Fig. 3D): pNbleb slightly increased the fluorescence recovery rate constants in the low concentration range, peaking at 7.5 µM (*k*_pNbleb,7.5_ = 7.5 × 10^−3^ ± 2.9 × 10^−3^ s^−1^), where the rate constants were statistically different from those in the untreated controls (3.1 × 10^−3^ ± 7.4 × 10^−4^) (Fig. 3C). Above 7.5 µM pNbleb, the rate constant of fluorescence recovery dropped significantly (Fig. 3C,D). Thus, strikingly, different concentrations of pNbleb leads to opposite effect on fiber dynamics, possibly due to different shifts in the proportion of weak and strong actin binding myosin states.

We also tested whether the diffusion rate of the inhibited NM2 is isoform-specific. FRAP experiments were carried out using HeLa cells transfected with NM2A or NM2B isoforms labelled with GFP on the N-terminus of their heavy chains (GFP-NM2A or GFP-NM2B). Fluorescence recovery of the photobleached peripheral stress fibers was monitored in both cell lines in the presence of different concentrations of pNbleb (5–20 µM). We found minor differences between the fluorescence recovery rate constants of the two isoforms. Also, we found that the response to pNbleb treatment followed the same trends as in the case of light chain GFP-labeled NM2 constructs (Table 1).

**Table 1:** Fluorescence recovery rate constants determined for different NM2 isoform-expressing cells and GFP-MLC cells.

[pNbleb]	*k*_GFP-MLC_ (s^−1^)		*k*_NM2A_ (s^−1^)		*k*_NM2B_ (s^−1^)	
	Mean	S.E	Mean	S.E	Mean	S.E
0 μM	4.3 × 10^−3^	± 8.9 × 10^−4^	8.3 × 10^−3^	± 1.7 × 10^−3^	8.6 × 10^−3^	± 3.3 × 10^−3^
5 μM	8.3 × 10^−3^	± 3.0 × 10^−3^	8.5 × 10^−3^	± 2.0 × 10^−3^	1.5 × 10^−2^	± 5.8 × 10^−3^
20 μM	5.2 × 10^−4^	± 1.7 × 10^−4^	1.1 × 10^−3^	± 1.9 × 10^−3^	1.0 × 10^−3^	± 1.6 × 10^−3^

The table shows the mean and the standard error (S.E.) of the recovery rates acquired from the cells expressing GFP-MLC (*k*_GFP-MLC_), NM2A heavy chain labeled myosin (*k*_NM2A_) and NM2B heavy chain labeled myosin (*k*_NM2B_). The rate constants show similar trends upon pNbleb inhibition in all three types of labeling methods.

### Subcellular localization of inhibitor effects achieved by Molecular Tattoo

**Figure 4:**
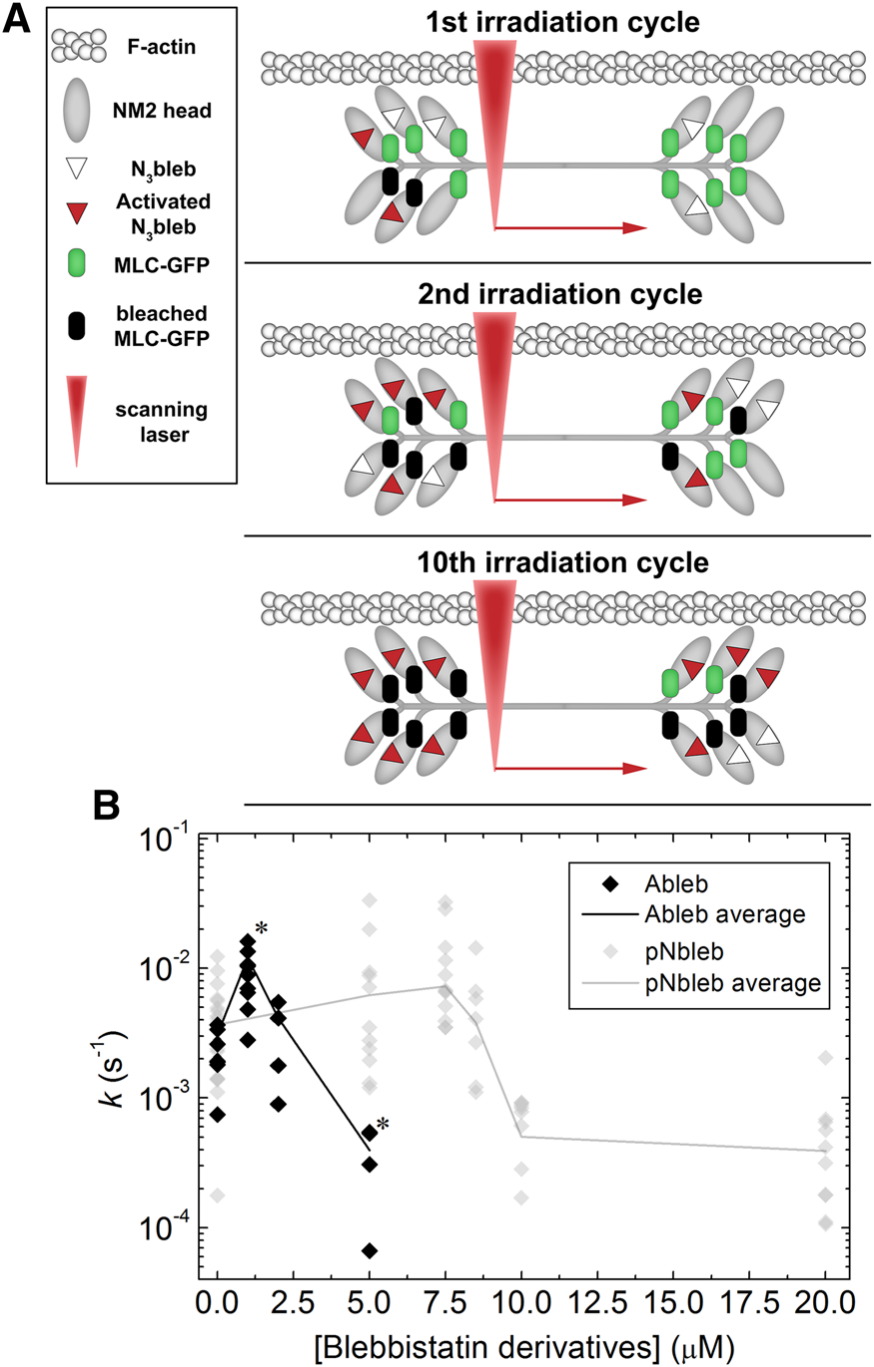
FRAP experiments on stress fibers locally inhibited by Molecular Tattoo. (**A**) Schematic of the combination of Molecular Tattoo with FRAP technique (FRAP-Tattoo). In FRAP-Tattoo, azidoblebbistatin (N_3_bleb) is photocrosslinked to its target protein, NM2, in a subcellular region, while simultaneously bleaching GFP attached to the myosin light chain (MLC) in the same subcellular area. Crosslinking and photobleaching are achieved using multiple irradiation cycles with a two-photon microscope at 860-nm excitation. Red arrow indicates the movement of the scanning laser (red triangle). N_3_bleb is added in sub-effective concentrations (1, 2 and 5 μM) to avert systemic NM2 inhibitory effect. Several irradiation cycles saturate the covalently crosslinked NM2 inhibitor population. Simultaneously, the GFP tag on the MLC is photobleached. (**B**) Fluorescence recovery rate constants in FRAP-Tattoo experiments as a function of N_3_bleb concentration (black diamonds) (n_0 μM_ = 7, n_1 μM_ = 9, n_2 μM_ = 4, n_5 μM_ = 5). Gray diamonds represent the rate constants obtained from Fig. 3D. Black and gray lines connect averages of rate constant values. The graph shows all acquired data points. Datasets marked with black asterisks are significantly different from control recovery rates (0 μM), according to parametric ANOVA tests (see “Materials and methods”).

We used the novel optopharmacological tool, Molecular Tattoo (MT)^43^, to localize the effect of blebbistatin inhibition to a small part of a single stress fiber. We sought to determine whether the non-monotonic dependence of FRAP rate constants on pNbleb concentration arose solely as an effect of inhibition of the peripheral stress fibers or other, non-inhibited regions also affect NM2 diffusion. MT enables the photocrosslinking of azidated inhibitors to target molecules (Fig. 4). Thus, even at sub-effective inhibitor concentrations, saturation of targets can be achieved in a confined subcellular area^43^. We used MT to covalently attach a photoreactive azidated derivative of blebbistatin, azidoblebbistatin (N_3_Bleb)^46^, to NM2 in selected areas of peripheral stress fibers.

MT was combined with the FRAP technique (FRAP-Tattoo), by applying multiple irradiation cycles, to bind N_3_Bleb to NM2, with concomitant bleaching of the GFP signal of the peripheral stress fibers with the two-photon laser (Fig. 4A). In the FRAP-Tattoo tests, FRAP recovery rate constants were determined as a function of N_3_Bleb concentration (1–5 µM, Fig. 4B). Similarly to previous experiments, we evaluated the fluorescence recovery of peripheral stress fibers at the tattooed areas by fitting single exponential functions. Interestingly, when the rate constants of the individual measurements were plotted as a function of inhibitor concentration, we observed the same phenomenon as with pNbleb but at markedly lower concentrations (Fig. 4B). To eliminate the possibility that our results were artefacts resulting from non-specific reactions caused by irradiation (e.g. photo-crosslinking of N_3_Bleb with off-target proteins), we also used the inactive (+) stereoisomer of N_3_Bleb^46^. Fluorescence recovery rate constants in the presence of the inactive stereoisomer were the same as those in the absence of inhibitor (Supplementary Fig. 6), proving that the measured recovery rate profile was indeed the consequence of localized NM2 inhibition and not the result of aspecific reactions or photo-damage. Further control FRAP-Tattoo experiments were carried out in the presence of 5 μM of the active (–) N_3_Bleb stereoisomer but at 1,000 nm excitation wavelength where photocrosslinking is not induced but GFP photobleaching still occurs (Supplementary Fig. 6). We did not observe any significant difference in peripheral stress fiber dynamics compared to the untreated cells. The results of the FRAP-Tattoo experiments indicate that the inhibitory effect of blebbistatin on NM2 diffusion dynamics remains localized to the inhibited fibers and it is not affected by a cooperative effect between the inhibited and non-inhibited regions.

### Direct myosin inhibition does not affect central fiber diffusion rates

**Figure 5:**
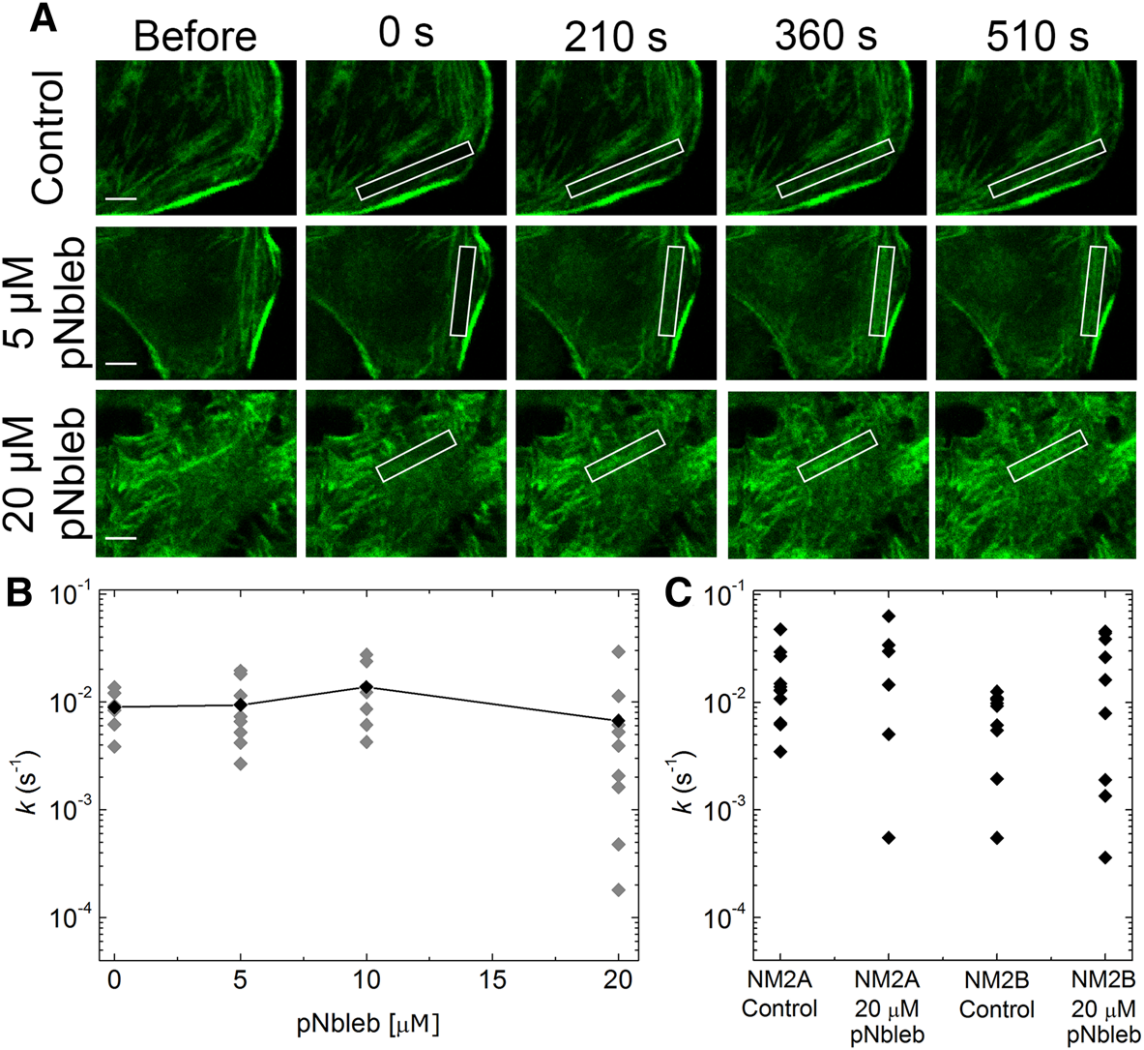
NM2 diffusion in central stress fibers is insensitive to pNbleb treatment and is NM2 isoform-independent. (**A**) Representative FRAP images of central fibers of GFP-MLC expressing HeLa Kyoto cells at different time points in the absence (upper row) and presence of 5 μM pNbleb (middle row) and 20 μM pNbleb (lower row). White boxes indicate photobleached areas. Scale bar: 5 μm. (**B**) Plot of FRAP rate constants (grey diamonds) as a function of pNbleb concentration. Averages of FRAP rate constants are shown as black diamonds connected with a black line (n_0 μM_ = 7, n_5 μM_ = 8, n_10 μM_ = 6, n_20 μM_ = 9). At 20 μM pNbleb concentration, the average of the rate constants is not significantly different from those at lower inhibitor concentrations, while the variation of the rate constants is higher (σ^2^_0μM_ = 1.1 × 10^−5^; σ^2^_10μM_ = 9.3 × 10^−5^ and σ^2^_20μM_ = 8.4 × 10^−5^), which may originate from the occasional strained state of the central fibers tested in these experiments. (**C**) FRAP rate constants of central fibers in GFP-NM2A or GFP-NM2A expressing cell lines in the absence and in the presence of 20 μM pNbleb (n_NM2A 0 μM_ = 12, n_NM2A 20 μM_ = 6, n_NM2B 0 μM_ = 11, n_NM2B 20 μM_ = 10). Rate constants did not differ significantly from those obtained for the GFP-MLC construct, and the increased variation of rate constants at 20 μM pNbleb was also observed for GFP-NM2 heavy chain isoforms.

We also tested the effect of pNbleb on NM2 diffusion in central fibers. According to the literature, the peripheral and central fibers may have different strain levels^17^, which could alter the actin binding properties of NM2 and therefore lead to different diffusion patterns^18–20^. FRAP experiments were carried out using HeLa Kyoto GFP-MLC cells (Fig. 5) and recovery was followed in central fiber structures in the presence of different pNbleb concentrations (0–20 µM, Fig. 5B). The FRAP recovery rate constants indicate that the diffusion of NM2 in the central fibers was ten times faster than that in the peripheral stress fibers in the absence of inhibitors. In the presence of either high or low concentrations of pNbleb, no significant effect on the fluorescence recovery rate constants of the central fibers was found; however, the variance of the recovery rate constants increased at 20 µM pNbleb (Fig. 5B) which may originate from the occasional straining of the otherwise mainly unloaded central fibers in the FRAP test (Figs. 2, 3). Similar results were found when heavy chain-labelled GFP-NM2A and GFP-NM2B were tested in the central fibers, indicating that NM2 diffusion is not isoform specific upon blebbistatin inhibition (Fig. 5C).

## Discussion

NM2A and NM2B isoforms are key components of stress fibers as they assemble into minifilaments connecting and moving actin bundles. The ATPase activity of NM2 can be inhibited by the ROCK-specific inhibitor Y-27632^33,34^ and blebbistatin^35,37–39^, although with different molecular mechanisms. ROCK inhibition leads to decreased phosphorylation levels of the regulatory MLC, which facilitates the formation of the 10S state^29^. Here we confirmed that the fluorescence recovery (FRAP) of peripheral stress fibers is greatly accelerated in the presence of Y-27632 (Fig. 3A,B)^47^, which can be explained by a shift in the equilibrium toward a highly mobile but enzymatically inactive state of NM2, which is probably also responsible for the disruption of the stress fibers (Fig. 1). It has been suggested that this dephosphorylated, highly mobile fraction of NM2 proteins in the cellular environment allows for faster displacement of NM2 molecules, which could not be attained via the active translocation of the NM2 assemblies^30–32^. Y-27632 treatment greatly increases the rate at which NM2 enters this fraction, to the level at which the stability of the stress fibers could also be compromised.

In contrast to the inhibition of ROCK, which is an upstream regulator of NM2, blebbistatin and its derivatives (AmBleb, pNbleb, N_3_Bleb) exert allosteric inhibition to block the ATPase activity of NM2 by directly binding to the myosin motor domain^48^. All blebbistatin derivatives used in this study retain similar inhibitory properties to those of blebbistatin^41,42,46^, while lacking the undesirable features of the original molecule^40^.

In the absence of inhibitors, simultaneous photobleaching of peripheral and central stress fibers yielded tenfold difference between the respective FRAP recovery rates, with central fibers showing more rapid dynamics (Fig. 2). Furthermore, at low concentrations pNbleb treatment increased the recovery rate until 7.5 µM, and beyond this concentration, the FRAP recovery decreased significantly (Fig. 3C,D, Supplementary Video 3, Supplementary Fig. 6). Strikingly, the FRAP rate constants of central fibers remained unaffected by inhibitor treatment both at low and high concentrations (Fig. 5A,B). We also successfully utilized Molecular Tattooing of N_3_bleb to NM2 in a confined subcellular region, which resulted in the same effect pattern on NM2 diffusion as systemic pNbleb treatment (Fig. 4). This finding indicates that the increased and decreased NM2 diffusion rates in the partially and fully inhibited NM2 populations, respectively, are caused by autonomous molecular processes.

The response of NM2 to different levels of external load is a mechanism by which FRAP rates could be altered, as this can greatly affect the actin binding properties of NM2 motor proteins. Structural investigations have shown that external load can disrupt the allosteric communication between the subdomains of the NM2 motor domain^49^, which in turn increases the lifetime of the strong-actin binding primed-lever state of myosin with bound ADP (M.ADP state), leading to NM2 spending longer time intervals bound to actin^18,49^. Tanner et al. have shown that significant regional differences are present in the stress fiber populations in regards to strain; namely, peripheral stress fibers showed greater retraction upon laser ablation compared to central fibers, suggesting higher levels of strain^17^. The one order of magnitude difference in the uninhibited FRAP recovery rates could originate from the different ratio of NM2 heads adopting the M.ADP and M.ADP.P_i_ states, which is due to the unequal strain acting on these stress fiber sub-populations. Furthermore, the M.ADP state has been shown to be also stabilized in the presence of blebbistatin, although this state has about 10 times lower affinity to blebbistatin than the M.ADP.P_i_ state^50^. The differential response to inhibitor treatment could also be a result of the strain-dependent ratio of M.ADP.P_i_ and M.ADP within the stress fiber sub-populations, as the preponderance of either state could alter the response to pNbleb treatment through their different actin affinities. Furthermore, differential strain acting on the stress fibers could be the reason why the central stress fibers showed only occasional response to high concentrations of pNbleb, leaving average recovery rates unaltered, while the variance in recovery rates increased significantly. This can be accounted for by the occasional straining of these structures, leading to varying response to inhibitor treatment. Within the boundaries of this study, we cannot confirm either the presence or the absence of differential strain, but there are indications that the strain sensing ability of NM2^49^ might be responsible for the distinct FRAP recovery rates elicited by pNbleb treatment.

In summary, our results highlight the consequences stemming from the differences between the molecular mechanism of NM2 ATPase inhibition through the upstream effector ROCK (with Y-27632) and that using direct NM2 inhibitors such as blebbistatin and its derivatives. Thus, in cell physiological experiments, Y-27632 and blebbistatin derivatives should not be considered as functionally equivalent forms of drug treatment. Furthermore, the effect of low and high blebbistatin concentrations must be considered separately, due to their discussed, opposing effect on peripheral stress fiber dynamics. Understanding the control of NM2-mediated fiber dynamics is of paramount importance as it plays essential roles in key physiological functions such as cell division, cell motility, neuronal morphology including axonal growth cone regulation, neurite outgrowth and axon initial segment plasticity^4,8–10,51,52^.

## Materials and methods

### NM2 heavy chain constructs

The NM2 heavy chains were cloned into pEGFP-C3 with a TET promoter (pTRE-GFP-NMHC II-A, Addgene ID: #10844, pTRE-GFP-NMHC II-B, Addgene ID: #10845)^53^. The NM2 heavy chain contructs were kindly provided by Robert S. Adelstein’s lab (National Heart, Lung and Blood Institute, Bethesda, MD, USA).

### MLC12B construct

The rat MLC12B construct, was expressed in HeLa Kyoto cells from the cytomegalovirus promoter using pEGFP-N1 vector^44^.

### Cell culture

HeLa Kyoto GFP-MLC cells, expressing GFP-labeled rat myosin light chain 12B (MLC12B)^44^ (generously provided by Daniel Gerlich and Felix Spira, IMBA Vienna) were cultured in DMEM (4.5 g/L glucose, with L-glutamine (Lonza)), supplemented with 10% fetal bovine serum (FBS; Gibco). GFP-MLC expression was maintained with Hygromycin b (Thermo Fisher) at 1.5 mg/mL. For the two-photon microscopic studies the cells were seeded on 1.0 borosilicate dishes (Mo-Bi-Tec) at a concentration of 1.4 × 10^5^ cells/mL. In FRAP experiments a medium lacking phenol-red was used (4.5 g/L glucose, without l-glutamine (Lonza)). This was further supplemented with 10% FBS and 25 mM HEPES (pH 7.3) buffer to achieve optimal environment for the cells. HeLa Original cells were maintained in low-glucose DMEM (1 g/L glucose with Glutamax (Gibco)), supplemented with 10% FBS. For transfection with the GFP-NM2A and GFP-NM2B constructs reported earlier^53^, cells were cultured on 1.0 borosilicate dishes at 1.4 × 10^5^ cells/mL. Lipofectamine 3000 reagent (Thermo Fisher) was used for transfection, which was carried out according to the protocol provided by the manufacturer (Thermo Fisher). The cells were incubated for an additional day to achieve higher GFP-NM2A and GFP-NM2B expression. The transfection medium was also exchanged with DMEM without phenol-red.

### Inhibitor treatment

para-Nitroblebbistatin (pNbleb) and azidoblebbistatin (N_3_Bleb) were synthesized by ChiroBlock GmbH. Aminoblebbistatin® (AmBleb; Patent ID: WO2017129782 (A1)) was prepared in our lab as described in^42^. The ROCK-specific inhibitor Y-27632 was purchased from Sigma-Aldrich. For FRAP studies the inhibitors were added to the cells in the imaging medium (DMEM without phenol-red) 1 h prior to the experiments.

### Two-photon microscopy

FRAP experiments were carried out using a custom two-photon microscope (Femtonics). The light source of the microscope is a mode-locked MaiTai Ti:Sapphire laser (Spectra Physics). GFP excitation was achieved using 900-nm wavelength with 1.75% (P_avg_ = 11.7 mW) intensity, while photobleaching of the fluorophore required tuning it to 8% (P_avg_ = 59.9 mW). Photobleaching was induced in a 1.5 × 0.2 µm area of a peripheral stress fiber. Fluorescence recovery was then followed for 10 min. Molecular tattooing (attachment of a photoreactive bioactive compound to its target within the highly confined focal spot during two-photon irradiation) of the peripheral stress fibers required multiple irradiations, to saturate NM2 with N_3_Bleb, while also bleaching the GFP signal in HeLa Kyoto GFP-MLC cells. A small area of the peripheral stress fiber was tattooed using 10 irradiation cycles each lasting 2 s, without delay between them, using 3.5% (P_avg_ = 25.2 mW) laser intensity at 860 nm (optimal wavelength for photo-cross linking with N_3_Bleb). Fluorescence recovery was monitored with 1.75% laser intensity at 900 nm for 10 min.

### Data analysis

Fluorescence recoveries were analyzed in single-section images with FIJI. Fluorescence was corrected for bleaching during imaging by using the fluorescence of a different peripheral stress fiber in the same cell. These data points were plotted as a function of time and fitted with exponential functions using Origin 8.5 (OriginLab). Statistical hypothesis testing was performed on log-transformed rate constant values using one-way ANOVA followed by Tukey’s *post-hoc* test (*p* < 0.05).

Peripheral stress fiber structure analysis was carried out using MATLAB (Release 2011b, The MathWorks, Inc.), which included the calculation and normalization of fluorescence intensity histograms using the following procedure on single section images of whole cells obtained with two-photon microscopy. (i) The background signal of the images (fluorescence outside cells) was subtracted and the fluorescence intensity distributions of whole cells were obtained. (ii) The intensity distribution was multiplied by pixel intensity. The intensity of a pixel is proportional to the local concentration of GFP-labeled NM2 light chains. Thus, the intensity-multiplied distribution is proportional to the amount of labeled myosin within a cell. (iii) The multiplied distribution was divided by the total intensity, which is proportional to the amount of observable NM2. The intensity distribution normalized in this way shows the proportional amount of MLC labeled myosin in the cell at a given intensity. (iv) In order to make cells with different size and myosin content comparable, the intensity scale of the distribution was normalized by its mean value. The intensity distributions of multiple cells were thus transformed and aggregated for each condition. Two sample t-tests were performed to assess the effect of the inhibitors on the pixel intensity distributions of the studied cells.

## Supplementary information

Supplementary Figures

Supplementary Video 1

Supplementary Video 2

Supplementary Video 3
