# Supplementary material for: Effect of allosteric inhibition of non-muscle myosin 2 on its intracellular diffusion
Source: Sci Rep. 2020 Aug 7;10:13341. doi: 10.1038/s41598-020-69853-8 (PMC7415145; doi:10.1038/s41598-020-69853-8)
Supplement: Supplementary file 1 — Supplementary Figures [file 41598_2020_69853_MOESM1_ESM.pdf]

Supplementary Information for "Effect of allosteric inhibition of non-muscle myosin 2 on its intracellular diffusion"

Ádám I. Horváth, Máté Gyimesi, Boglárka H. Várkuti, Miklós Képiró, Gábor Szegvári, István Lőrincz, György Hegyi, Mihály Kovács, András Málnási-Csizmadia

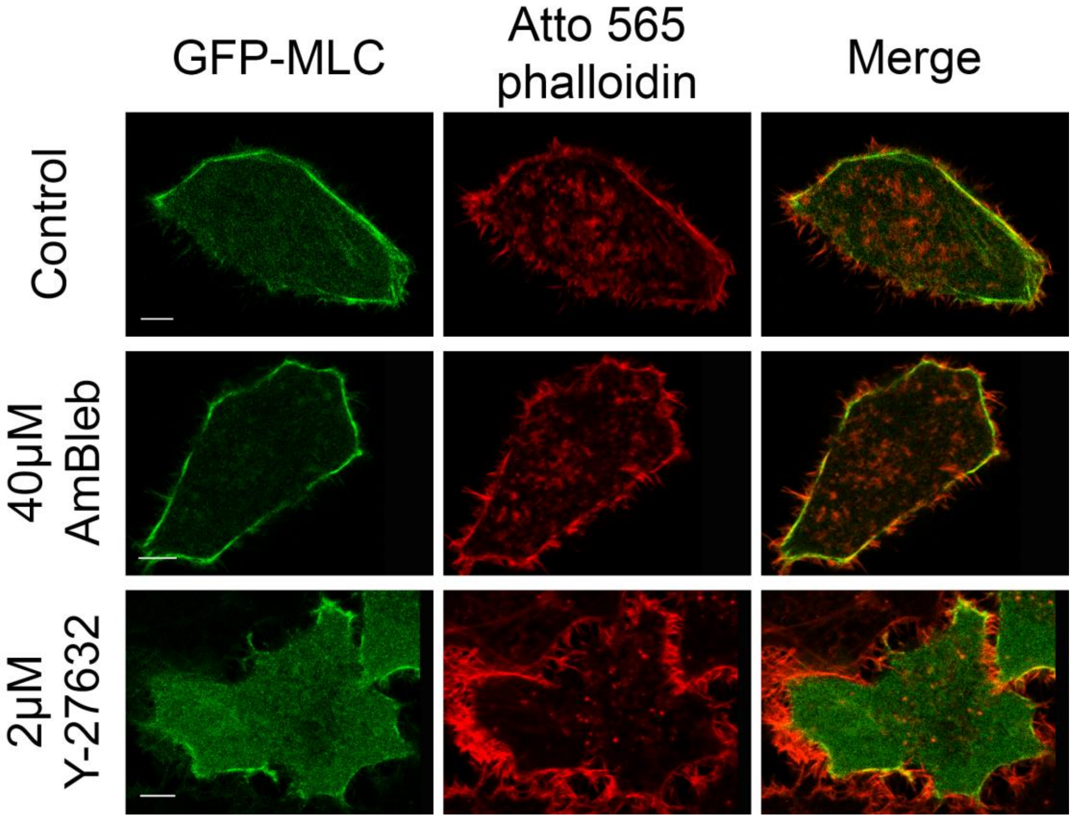

Supplementary Figure 1. Phalloidin staining of HeLa Kyoto GFP-MLC cells. Staining fixed HeLa Kyoto GFP-MLC cells with the actin specific dye, Atto 565-phalloidin, shows co-localization between the GFP-labeled NM2 light chains and the phalloidin-stained actin fibers, which act in concert to form the stress fibers in these cells. Furthermore, using the same pharmacological inhibition as in **Fig. 1**, the same effect can be elicited here. Namely, the ROCK specific inhibitor, Y-27632, causes partial dissolution of stress fibers and the GFP fluorescence is shifted to the inner regions of cells. Using AmBleb slightly increases the inner fluorescence, while maintaining stress fiber integrity.

HeLa Kyoto GFP-MLC cells were grown in monolayer (~90 % confluency) on 35-mm 1.0 dishes (Mo-Bi-Tec) using standard growth medium. After reaching the desired confluency, cells were washed twice with 37°C PBS. The cells were fixed for 10 min using freshly made 4 % para-formaldehyde (PFA, Sigma-Aldrich) solution (in PBS). PFA was removed by washing the cells tree times with PBS. Cells were permeabilized with 1% Triton X-100 for 5 minutes. The detergent was removed by washing the cells with PBS three times. Atto phalloidin (Sigma-Aldrich) stock solution (20 µM) was diluted to a final concentration of 400 nM with PBS. Cells were incubated for 30 minutes in staining solution. After incubation, the staining solution was removed by washing the cells twice with PBS. Fluorescence images from both red and green channels were collected using a Zeiss LSM800 laser scanning microscope.

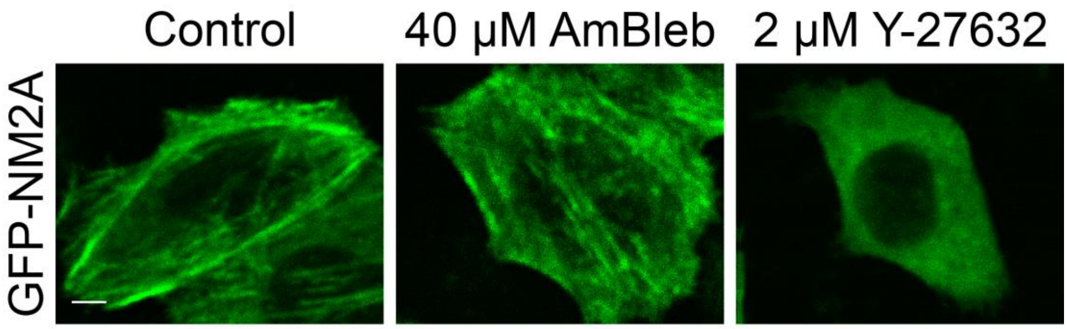

Supplementary Figure 2. GFP-NM2A heavy chain labeled HeLa cells with different stress fiber affecting inhibitors. Differential pharmacological inhibition elicits the same effect as seen in the myosin light chain labeled GFP-MLC cells. The myosin 2-specific AmBleb slightly disrupts the stress fiber integrity, while the Rho-kinase-specific Y-27632 causes the dissolution of both peripheral and central stress fibers.

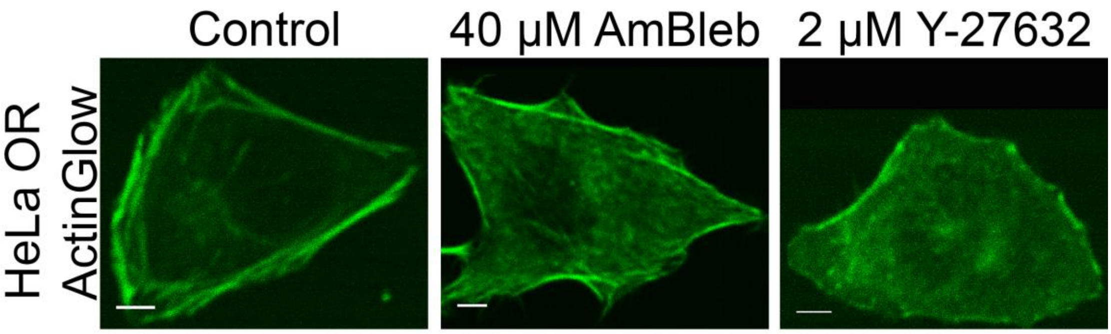

Supplementary Figure 3. ActinGlow stained HeLa cells under differential pharmacological inhibition. GFP label free HeLa OR cells stained with the actin-specific dye ActinGlow show minor effect on the actin structure upon AmBleb treatment, compared to untreated cells. Applying Y-27632 to these cells reduces the number of intact actin stress fibers.

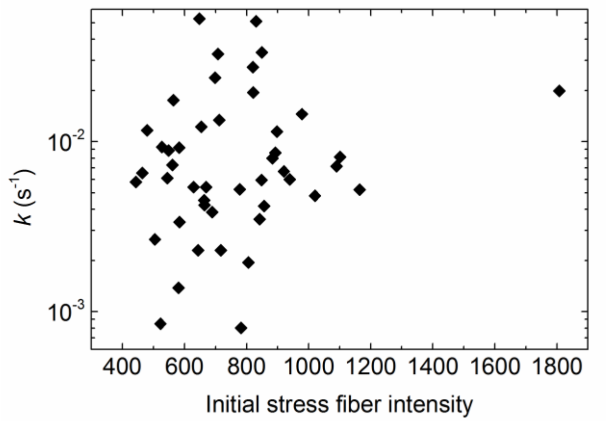

Supplementary Figure 4. FRAP rate constants as a function of initial stress fiber intensity. The rate constants in FRAP experiments performed in this study were plotted against the initial, un-bleached stress fiber intensities. The graph contains rate constants from both peripheral and central stress fiber experiments. The graph shows no correlation between the rate constant and the initial intensities of the FRAP-ed stress fibers (Pearson's correlation coefficient: 0.12).

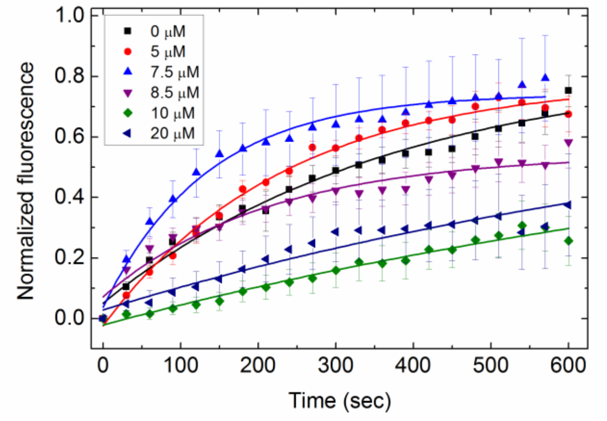

Supplementary Figure 5. Single exponential fits of averaged fluorescence intensities. Averaged fluorescence recovery of peripheral stress fibers is shown, recorded in the presence of 0, 5, 7.5, 8.5, 10 μM and 20 μM pNbleb. Single exponential fits are shown as black, red, blue, violet, green and navy blue lines, respectively.

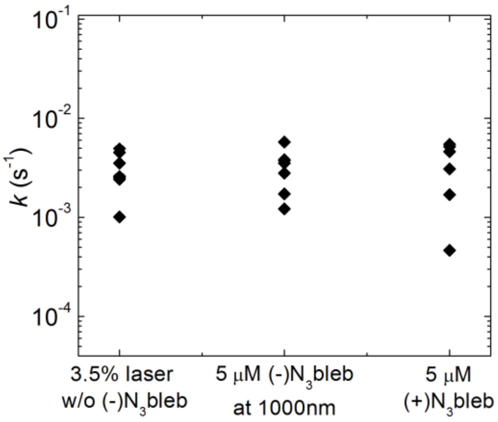

Supplementary Figure 6. Molecular Tattoo control experiments to exclude non-specific effects of N_3bleb crosslinking. FRAP rate constants are shown for control experiments in the absence and presence of 5 µM (-)N_3bleb (active enantiomer) at 1000-nm excitation wavelength (where no photocrosslinking occurs) and in the presence of 5 µM (+)N_3bleb (inactive enantiomer).

Supplementary Video 1
Simultaneous FRAP of the peripheral and central stress fibers

Supplementary Video 2
Low magnification video of the simultaneous FRAP of the peripheral and central stress fibers

Supplementary Video 3
FRAP experiments of the cells treated with 0 µM, 7.5 µM and 10uM pNbleb
